# High Risk Infants Follow-Up: A Case Study in Iran

**DOI:** 10.1155/2015/817540

**Published:** 2015-06-04

**Authors:** Mohammad Heidarzadeh, Behzad Jodeiry, Mohammad Baqer Hosseini, Kayvan Mirnia, Forouzan Akrami, Abbas Habbibollahi, Sara Moazzen, Saeed Dastgiri

**Affiliations:** ^1^Pediatric Health Research Center, Tabriz University of Medical Sciences, Tabriz 5166615739, Iran; ^2^Medical Ethics and Law Research Center, Shahid Beheshti University of Medical Sciences, Tehran, Iran; ^3^Deputy of Public Health, Ministry of Health and Medical Education, Tehran, Iran; ^4^Tabriz Health Services Management Research Centre, Tabriz University of Medical Sciences, Tabriz 5166615739, Iran

## Abstract

*Background*. A follow-up program for high risk infants was initiated in Alzahra Maternity Hospital in Tabriz city, Iran, in 2013. The aim of this paper is to give a brief report of the program. *Material and Methods*. Two groups of high risk neonates were studied. The first group comprising 509 infants received services in Alzahra Maternity Hospital implemented by the follow-up program. This included a full package for family to look after high risk infant and periodic clinical evaluation at two and four weeks after birth and then two, three, four, five, and six months later again. The second group including 131 infants in Taleqani Maternity Hospital received routine services after birth with no specific follow-up care. *Results*. Some anthropometric indices showed a significant improvement in the intervention hospital compared to control group. These included the following: head circumference at first and second months; weight in the first, fourth, fifth, and sixth months; and height in sixth month only. Clinical evaluation of infants showed an improvement for some of the medical conditions. *Conclusion*. Follow-up care program for a minimum of six months after discharge from maternity hospitals may help to avoid adverse and life threatening consequences in high risk infants.

## 1. Introduction

According to Barker's theory, certain risk exposures few months before pregnancy, during pregnancy, and in early childhood might have a role in the occurrence of some disorders in adulthood [1]. It is, therefore, essential to monitor the medical status of neonates, particularly those at certain risks, to keep them healthy and to avoid adverse consequences later in the life. Some studies showed that for preventing irreversible consequences in high risk infants, they need to be specifically looked after by a team of experts with various skills [2–12]. The definition of high risk infants and the approaches suggested for the follow-up of high risk children are discussed in detail elsewhere [12]. Implementation of any follow-up program for high risk infants would largely depend on existing facilities, family circumstances, and infant status. [13–18].

A follow-up program for high risk infants was initiated in Alzahra Maternity Hospital in Tabriz city of Iran in 2013 [19]. The main objective of the program was to create a pilot model for possible implementation of follow-up care for high risk infants across the country. The aim of the current paper is to give a brief report of this program.

## 2. Material and Methods

This study was carried out in Tabriz city. The city is one of the three major cities in Iran located in the northwest of Iran, a cold climate zone, with a total population of 1,398,060 in 2012 and an average annual population growth rate of 0.8 percent.

High risk infant was mainly defined based on the birth weight (less than 1500 grams) and gestational age (less than 32 weeks). If an infant did not, however, have those criteria, other medical conditions (i.e., major surgery in the neonatal period, seizures or fits, and serious illnesses such as meningitis or congenital malformation) were considered for risk assessment at birth/soon after. Full details of the high risk conditions can be found elsewhere [20, 21].

Two groups of high risk neonates were studied. The first group comprising 509 infants (282 boys and 227 girls) received services in Alzahra Maternity Hospital implemented by the follow-up program. This included a full package for family/mothers to train them to look after high risk infant and periodic clinical evaluation by fully equipped/trained medical staff at two and four weeks after birth and then two, three, four, five, and six months later again. At those time points, the pediatric nurse of the program called the family (mainly mother) to bring the child for clinical follow-up. At this regular medical program, every infant was assessed for cardiac problems, lung diseases, gastroesophageal reflux, anemia and nutritional assessment (mainly based on weight, height, and head circumference), immunization (according to the country regular plan), hypothyroidism, osteopenia, nephrocalcinosis, renal tabular acidosis, Developmental Dislocation of Hip (DDH), intraventricular hemorrhage, neurodevelopmental assessment (using Ages and Stages Questionnaire at the second, fourth, and sixth months), retinopathy, auditory assessment, a comprehensive psychological assessment and mental health (including autism, etc.), sleep disorders (using a standardized questionnaire), oral health assessment, and anthropometric indices (including weight, height, and head circumference). Bronchopulmonary Dysplasia (BPD) is routinely fully managed during the hospital stay for every high risk infant. After discharge, a detailed follow-up program is organized beginning from the fourth year onward.

The second group including 131 infants (63 boys and 68 girls) in Taleqani Maternity Hospital received routine services after birth with no specific/regular follow-up or any specific nurse for this. They were medically assessed at the same time points and at the same hospital to compare them with the status of high risk infants in the first group. Infants diagnosed, as high risk infants, in two maternity centers (Alzahra, as pilot, and Taleqani, as control hospitals) entered the follow-up program. These hospitals routinely provide obstetric and gynecological services in the study population under the Tabriz University of Medical Sciences. Those hospitals are both public maternity facilities in similar social/economic areas. They are located in two different places in the city covering the population in two various zones in the city. Confounding factors were considered in both groups for the purpose of data analysis. Those factors included gender and risk status (based on birth weight and gestational age).

We also assessed the attitude and practice of staff and families towards the program using a questionnaire and interview and filling a check list while they are doing the job.

For the data analysis, we calculated 95% confidence interval for statistical indicators.

The study obtained ethics approval from the committee of ethics in Tabriz University of Medical Sciences. All personal and identity information were kept confidential. Medical staff and families were asked whether or not they are willing to participate in the study and were told that they are free to leave for any reason at any time.

## 3. Results

Of those high risk infants eligible for follow-up care in Alzahra Hospital, 33 percent (95% confidence intervals: 28.0–37.9) refused, even after they were called for three times, to enter the follow-up program where 84 percent (95% confidence intervals: 76.6–91.5) of control families with high risk infants did not participate in the hospital for follow-up services. In both groups, those refusing families either preferred to get follow-up services from private doctors and clinics or never got follow-up care at all over the study period.

We compared the improvement of clinical and anthropometric indices in Alzahra and Taleqani Hospital to assess the effectiveness of this program. Some anthropometric indices showed a significant improvement in the intervention hospital compared to control group over the follow-up period. These included the following: head circumference at first and second months; weight in the first, fourth, fifth, and sixth months; and height in sixth month only (Table 1). There was an overall improvement for the mean of head circumference, height, and weight between second week and sixth month (follow-up period) in Alzahra Hospital compared to control hospital. This change was statistically significant for mean height only.

Table 2 presents the proportion of high risk infants admitted to the outpatient clinics in both hospitals for assessment for various medical conditions and reasons. Clinical evaluation of infants in two hospitals broadly showed an increase in the screening for many of medical conditions. For instance, 21.6 percent of gastroesophageal reflux cases (95% confidence intervals: 18.0–25.2) were found and then medically managed in Alzahra Hospital where the same figure was 7.6 percent (95% confidence intervals: 3.1–12.2) in control hospital. In contrast, in some instances there was a better screening in control hospital: 14.5 percent of cardiac problems (95% confidence intervals: 8.5–20.6) were diagnosed in control hospital while it was 2.2 percent only in the first group (95% confidence intervals: 0.81–3.4).

## 4. Discussion

In this paper, we briefly reported a follow-up care program for high risk infants in Tabriz city of Iran. We showed that the program was seemingly effective in finding and managing some of the medical conditions in neonates over the six-month follow-up. Similar findings were reported before from various countries [22–28]. However, for implementing the program at regional or national levels, some technical, logistic, and financial aspects should be considered including the following:

- Before the program begins, it is essential to appoint a neonatologist (where unavailable, a general pediatrician) and a trained pediatric nurse/midwife as the focal points for the program in every hospital qualified for starting the follow-up care program for high risk infants. The program nurse/midwife will
  - coordinate the follow-up care with family;
  - provide the family with full package of the follow-up care (i.e., time table for clinical visits and instruction for various conditions and situations);
  - organize the timetable of clinical visits at regular basis;
  - arrange instruction and training sessions for family;
  - enter the relevant data in the program online software;
  - prepare regular reports of the follow-up program.
- Access to some paramedical services has to be organized/arranged for those infants needing these services after discharge. Some of those services include occupational therapy, physiotherapy, speech therapy, clinical nutrition services, and psychology and psychotherapy facilities. Some families may also need financial aids to access those services.
- A major attention should be made for recall and follow-up of those high risk infants living in the villages far from the program hospital. They may need several calls to come for follow-up care. The program nurse/midwife may also need to be in close contact with the local family doctor to organize the regular follow-up visit for those infants.

One of our main limitations in the implementation of this follow-up program was improper infrastructure and insufficient capacity of online data handling in the country. If a program is to be implemented across the country, the online communication of the data between states, hospitals, and main office in the ministry of health has to be set up/administered properly.

As a conclusion, a follow-up care program for a minimum of six months after discharge from maternity hospitals may help to avoid adverse and life threatening consequences in high risk infants.

## Figures and Tables

**Table 1:** Comparison of anthropometric indices in two hospitals.

	Alzahra Hospital (follow-up care)				Taleqani Hospital (control)			
	Mean	Standard deviation	Median	(95% confidence intervals)	Mean	Standard deviation	Median	(95% confidence intervals)
Head circumference (second week)	30.6	1.5	30.0	30.2, 31.0	32.3	2.5	32.3	31.7, 32.8 (^*∗*^)
Height (2 weeks) (second week)	42.4	2.5	42.0	41.6, 43.1	45.4	4.5	45.0	44.4, 46.7 (^*∗*^)
Weight (2 weeks) (second week)	1797.0	267.0	1735.0	1717.0, 1876.0	2259.0	756.0	2000.0	2084.0, 2435.0 (^*∗*^)
Head circumference (first month)	31.5	1.6	31.0	31.1, 31.9	33.5	2.7	33.0	32.8, 34.2 (^*∗*^)
Height (first month)	44.4	3.1	44.0	43.7, 45.1	46.5	5.8	46.0	45.4, 48.4
Weight (first month)	2122.0	429.5	1950.0	2022.0, 2222.0	2655.0	1004.0	2500.0	2400.0, 2910.0 (^*∗*^)
Head circumference (second month)	33.5	2.3	34.0	32.9, 34.1	35.2	2.3	35.0	34.5, 35.8 (^*∗*^)
Height (second month)	47.8	4.2	48.0	46.8, 48.8	48.9	5.3	48.0	47.0, 50.0
Weight (second month)	2911.0	873.0	2750.0	2698.0, 3124.0	3200.0	965.0	3050.0	2929.0, 3472.0
Head circumference (third month)	35.7	2.8	36.0	34.8, 36.6	37.0	2.3	37.0	36.3, 37.7
Height (third month)	52.9	9.1	52.0	49.9, 55.9	52.5	5.0	52.0	50.9, 54.0
Weight (third month)	3979.0	1683.0	3900.0	3417.0, 4540.0	4155.0	1317.0	3990.0	3745.0, 4566.0
Head circumference (fourth month)	37.2	2.0	37.0	36.5, 37.9	38.8	1.8	39.0	38.0, 39.5 (^*∗*^)
Height (fourth month)	54.8	4.7	55.0	53.2, 56.4	55.9	4.8	55.0	53.9, 57.9
Weight (fourth month)	4428.0	1037.0	4340.0	4077.0, 4778.0	5134.0	1140.0	5000.0	4672.0, 5593.0
Head circumference (fifth month)	39.0	2.5	40.0	37.6, 40.4	40.3	1.8	40.0	39.2, 41.4
Height (fifth month)	59.2	3.5	59.0	57.3, 61.1	57.8	6.3	57.0	53.9, 61.6
Weight (fifth month)	5512.0	695.0	5500.0	5127.0, 5898.0	5954.0	1534.0	6000.0	5027.0, 6881.0
Head circumference (sixth month)	41.3	2.2	41.0	40.8, 41.8	41.3	2.4	41.0	39.9, 42.6
Height (sixth month)	63.3	4.5	64.0	61.4, 65.2	59.5	3.4	60.0	57.6, 61.4 (^*∗*^)
Weight (sixth month)	6435.0	1250.0	6575.0	5963.0, 6907.0	6033.0	879.0	6000.0	5546.0, 6520.0

Change between second week and sixth month								
Head circumference	10.7		11.0		9.0		8.7	
Height	21.0		22.0		14.1		15.0	(^*∗*^)
Weight	4638.0		4840.0		3774.0		4000.0	

(^*∗*^)The difference between means is statistically significant.

**Table 2:** Proportion of high risk infants clinically managed over the follow-up period.

	Alzahra Hospital (follow-up care) (*n* = 509)		Taleqani Hospital (control) (*n* = 131)	
	Admitted/managed	Confidence intervals	Admitted/managed	Confidence intervals
	%	(95%)	%	(95%)
Cardiac problems	2.2	0.8, 3.4	14.5	8.5, 20.6 (^*∗*^)
Blood pressure	0	0.0, 0.0	0	0.0, 0.0
Gastroesophageal reflux	21.6	18.0, 25.2	7.6	3.1, 12.2 (^*∗*^)
Bronchopulmonary dysplasia	0.0	0.0, 0.0	0.0	0.0, 0.0
Respiratory syncytial virus	0.2	−0.1, 0.6	0.0	0.0, 0.0
Hypothyroidism	9.4	6.9, 11.9	18.3	11.7, 24.9
Osteopenia	5.3	3.4, 7.3	0.0	0.0, 0.0
Nutritional assessment	0.0	0.0, 0.0	17.6	11.0, 24.1
Nephrocalcinosis	2.2	0.8, 3.4	11.5	5.9, 16.9 (^*∗*^)
Renal tabular acidosis	0.0	0.0, 0.0	2.3	−0.3, 4.9
Anemia	19.6	16.2, 23.1	3.1	0.1, 5.9 (^*∗*^)
Developmental dislocation of hip	0.8	0.0, 1.6	0.0	0.0, 0.0
Oral health assessment	0.0	0.0, 0.0	0	0.0, 0.0
Autism	0.0	0.0, 0.0	0	0.0, 0.0
Child abuse	0.0	0.0, 0.0	0	0.0, 0.0
Sleep disorders	19.3	15.8, 22.7	9.2	4.2, 14.1 (^*∗*^)
Intraventricular hemorrhage	0.0	0.0, 0.0	0.8	−0.1, 2.3
Retinopathy	2.8	1.3, 4.2	0.8	−0.1, 2.3
Neurodevelopmental assessment	8.3	5.9, 10.7	0.0	0.0, 0.0
Auditory assessment	1.4	0.4, 2.4	0.0	0.0, 0.0

(^*∗*^)The difference between proportions is statistically significant.
